# Spontaneous Lymph Flow Restoration in Free Flaps: A Pilot Study

**DOI:** 10.3390/jcm12010229

**Published:** 2022-12-28

**Authors:** Théo Sedbon, Arié Azuelos, Romain Bosc, Francesco D’Andrea, Rosita Pensato, Michele Maruccia, Jean Paul Meningaud, Barbara Hersant, Simone La Padula

**Affiliations:** 1 Department of Plastic, Reconstructive and Maxillo Facial Surgery, Henri Mondor Hospital, University Paris XII, 51 Avenue du Maréchal de Lattre de Tassigny, 94000 Créteil, France; 2 Department of Plastic and Reconstructive Surgery, Università Degli Studi di Napoli Federico II, Via Pansini 5, 80131 Napoli, Italy; 3 Unit of Plastic, Reconstructive Surgery and Burn Center, Department of Emergency and Organ Transplantation, University of Bari Aldo Moro, 70124 Bari, Italy

**Keywords:** lymph flow restoration, free flaps, microsurgery, ALT flap, DIEP flap

## Abstract

Background: Oncologic excision and trauma can be responsible for major defects and lymphedema. Free flaps are commonly used for reconstruction. We aimed to determine if lymphatic flow between flap and recipient site can be restored without lymphatic surgery. Methods: 15 free flaps were performed in different patients in our center. Infrared-based lymphography was used to plan surgery. Indocyanine green (ICG) was injected in the flap’s subdermal tissue and also at the edges of the skin defect. Circumferential lymphatic channels were marked 5 min after the ICG injection. Fluorescent images were recorded with an infrared camera system. The flap inset was obtained by putting side to side the flap markings and the recipient site markings. Infrared-based lymphography was performed on every patient one year after surgery. Spontaneous lymph flow restoration was judged positive if lymphatic connections were observed between the flap and the recipient site. Results: seven free ALT and eight DIEP flaps were performed. All ALT flaps were designed following the limb axis which is the lymphatic axiality. Spontaneous lymph flow restoration was observed for the seven ALT flaps. Eight DIEP flaps were designed upside down and one was designed following the lymph axiality. Spontaneous lymph flow restoration was only observed for the one designed following the lymph axiality. Conclusions: designing reconstructive free flap regarding lymph axiality seems to improve spontaneous lymph flow restoration between flap and recipient site without any specific lymphatic surgery.

## 1. Introduction

The superficial lymphatic system has a parallel anatomic organization to the blood system. Its chronical obstruction, traumatism or excision can lead to chronic lymphedema impairing fluid balance and local immune reaction [1]. Major trauma and oncological excision associated or not to a node dissection often involves major lymphatic pathways [2,3,4,5]. Usually, lymphoangiogenesis occurs after trauma or excision and lymphatic pathways and functions are restored but some patients can develop lymphedema. Free flaps are a major asset for reconstructive procedures after oncologic excision and trauma [6,7,8]. Except for a few flaps like chimeric Superficial Circumflex Iliac Artery Perforator (SCIP) flaps or modified Deep Inferior Epigastric Perforators (DIEP) flaps involving inguinal lymph nodes, free flaps used for reconstructive surgery do not involve lymphatic tissue [9,10,11,12,13]. Lymphoangiogenesis and spontaneous lymphatic anastomosis to the recipient area lymphatic channels of these reconstructions are poorly described. Infrared-based lymphography using indocyanine green (ICG) is a routine examination for precise evaluation of the lymph system [14,15,16,17,18,19,20,21,22,23,24,25,26]. In this pilot study, we aimed to describe the spontaneous restoration of lymphatic flow of free flaps by infrared lymphography.

## 2. Materials and Methods

Between November 2019 and May 2020, 15 free flaps were performed in our center.

All patients were informed about the study and signed a written informed consent. Pre-operative lymphatic planning was performed in all cases. About 0.1 to 0.2 mL of indocyanine green (Infracyanine 25 mg/10 mL, Serb, Paris, France) was injected in the flap’s subdermal tissue and at the edges of the skin defect [24,27]. Circumferential lymphatic channels were marked 5 min after the ICG injection. Fluorescent images were recorded with an infrared camera system (Photodynamic Eye; Hamamatsu Photonics K.K., Hamamatsu, Japan). The flap inset was obtained by putting side to side the flap markings and the recipient site markings.

Infrared-based lymphography was performed one year after surgery in outpatient clinic. About 0.1 to 0.2 mL of indocyanine green (Infracyanine 25 mg/10 mL, Serb, Paris, France) was injected in the flap’s subdermal tissue, at the four extremities of the flap (proximal, distal, medial and lateral).

After the indocyanine green injection, fluorescent images of lymphatic drainage channels were obtained using an infrared-based camera system (Photodynamic Eye; Hamamatsu Photonics K.K., Hamamatsu, Japan). Indocyanine green lymphographic images were recorded immediately after the indocyanine green injection and 6 h after the indocyanine green injection.

Injections sites were checked on the early lymphography. If an injection was accidentally performed on the recipient site, the case was excluded.

Spontaneous lymph flow restoration was judged positive if linear patterns as described by Yamamoto et al. were observed between the flap and the recipient site and was judged as negative if no indocyanine green drainage was observed. This prospective observational study was approved by the French institution’s ethical committee with the number 2022-A02532-41.

## 3. Results

Some interesting results emerged from this preliminary pilot study.

**Figure 1:**
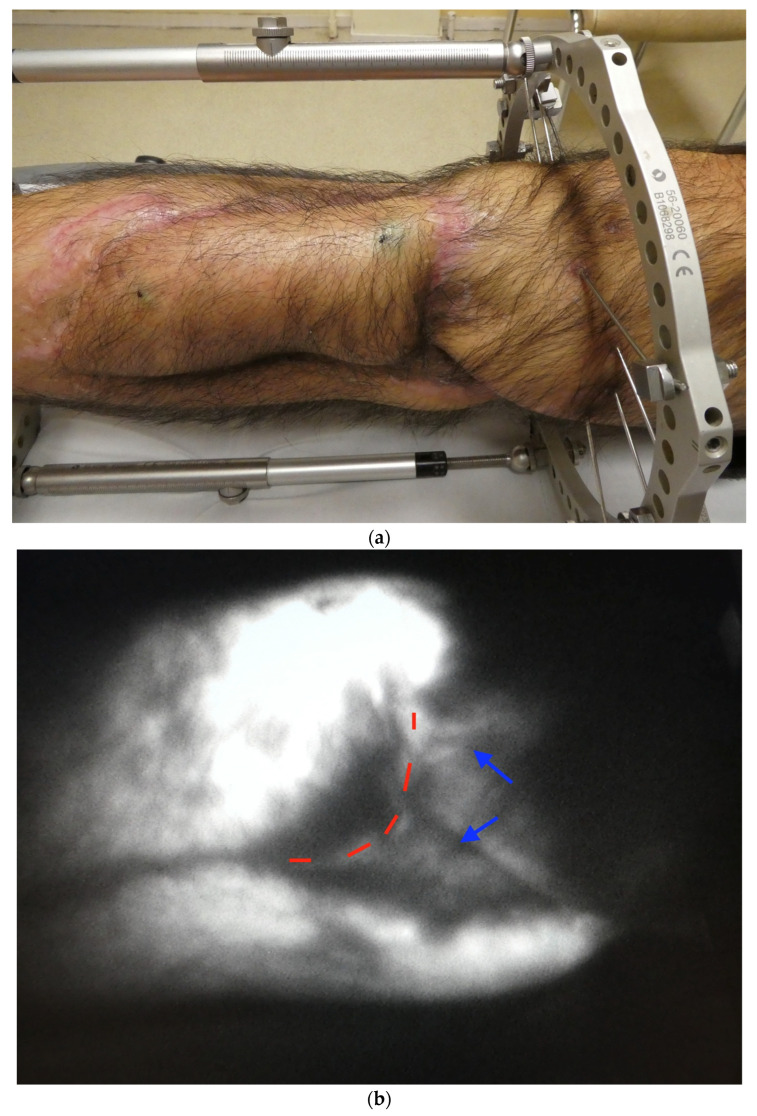
(**a**) Post-traumatic case, one year after ALT flap leg reconstruction. (**b**) ICG. Lymphatic channels (blue arrows) proximal to the flap (red dotted line).

**Figure 2:**
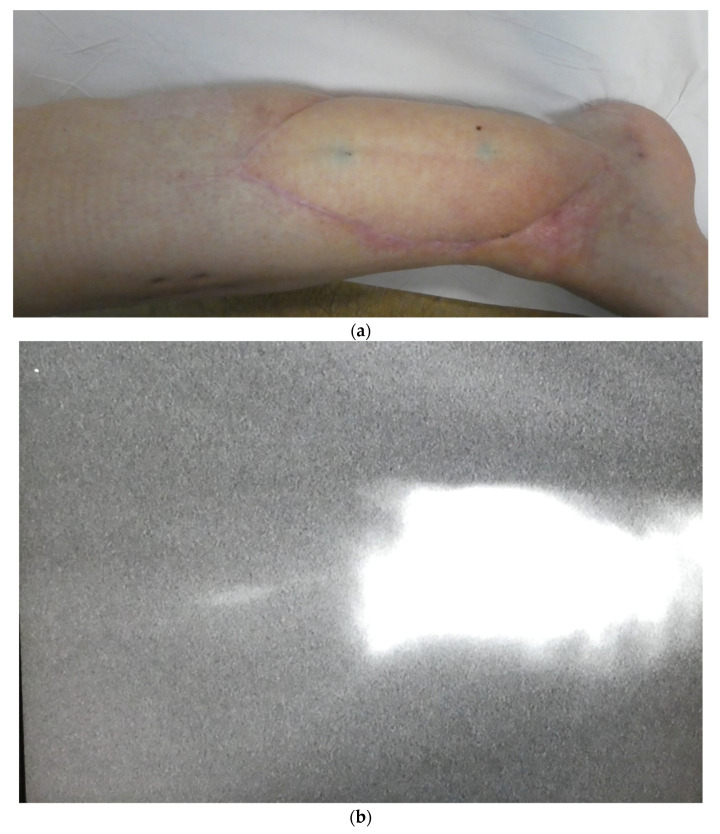
(**a**) Post-traumatic reconstruction with ALT flap using ICG guided flap design (one-year post-operative aspect). (**b**) ICG. Lymphatic connection between the flap and the recipient site is visible at the one year follow-up.

**Figure 3:**
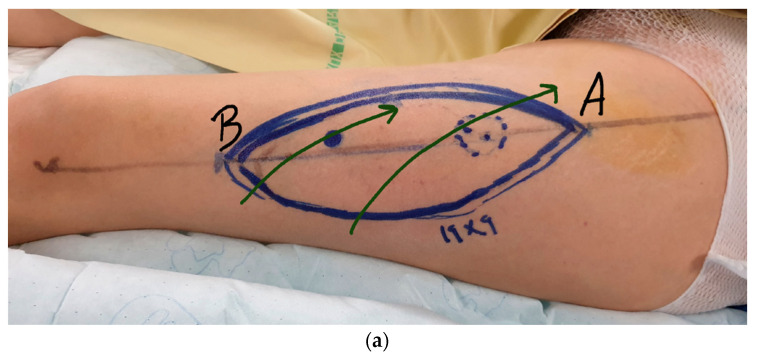

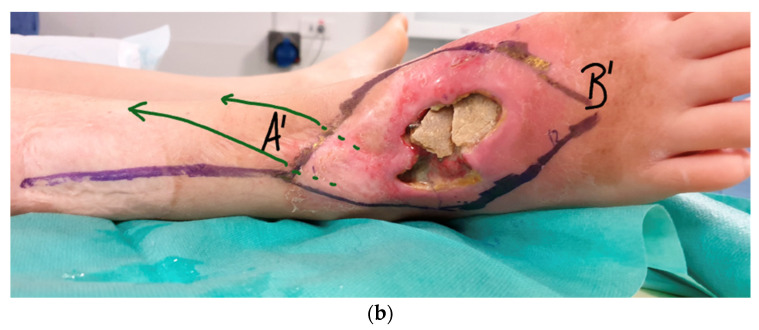
(**a**) An example of ALT flap pre-operative planning. (**b**) Cutaneous radionecrosis. ALT flap-based reconstruction was planned. Green arrows show the direction of lymphatic pathways. The A extremity of the ALT will be inserted in the A’ extremity of the defect and the B extremity of the ALT flap will be inserted in the B’ extremity of the defect to respect the lymphatic direction.

During a 6-month period, 7 free Anterolateral Thigh ALT flaps and 8 DIEP flaps were performed in our center. No case was excluded. ALT flaps were performed for limb reconstruction on five men and two women. Causes of the defect were acute traumatism secondary to public road accident for six patients. One patient suffered from tissue loss due to skin necrosis secondary to ankle osteosarcoma operative site infection. All ALT flaps were used for knee, leg or ankle reconstructions. ALT flaps were designed following the limb axis respecting the lymph flow axiality pathways. Moreover, the proximal extremity of each flap was placed in the proximal part of the defect on the limb. The flap was inserted into the defect in such a position that the lymphatic vessels axiality could be respected and approximated, as described by Yamamoto et al. and Shinaoka et al. [28,29]. For all the flaps, ICG preparative planning was used. Spontaneous lymph flow restoration was recorded for the seven ALT flaps. The spontaneous lymphatic anastomosis was observed between the transferred lymphatic vessels and the limb major lymphatic pathways. Two cases of post-traumatic reconstruction needed a secondary bone graft at 8 and 10 weeks after the reconstructive surgery. Incisions were placed avoiding major lymphatic patterns. No case of secondary clinically evident flap lymphedema was reported (Figure 1, Figure 2 and Figure 3).

**Figure 4:**
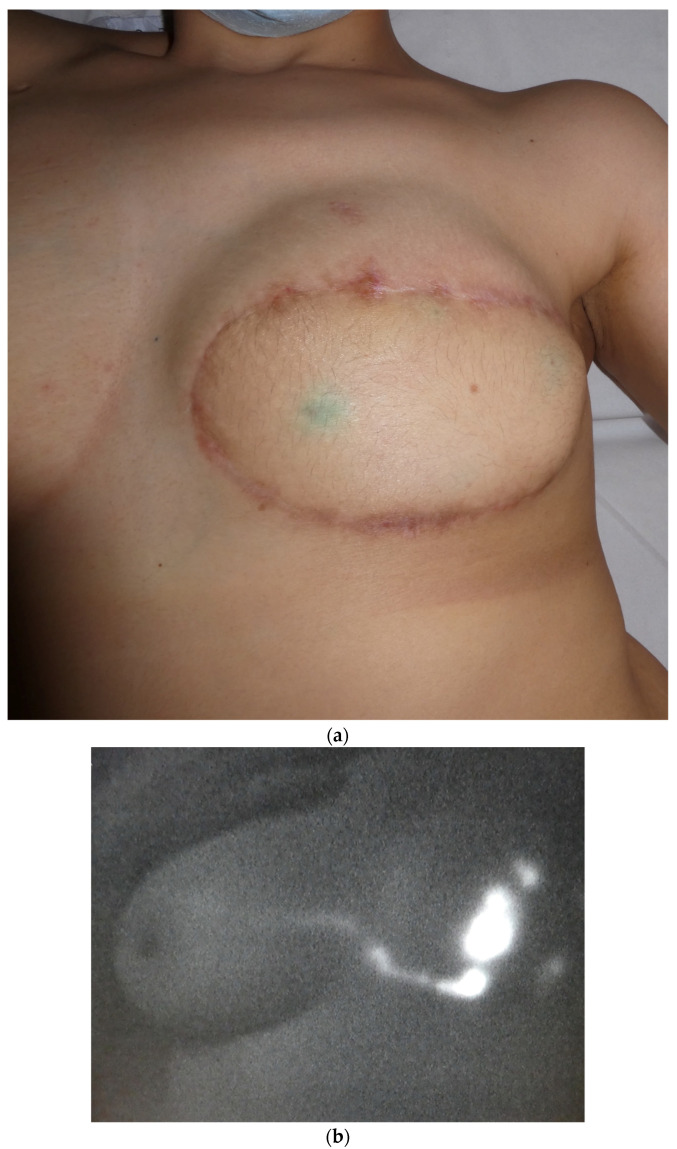
(**a**) Breast reconstruction with DIEP flap at one-year follow-up. (**b**) Breast reconstruction with DIEP flap and spontaneous lymph flow restoration at the one-year follow-up.

**Figure 5:**
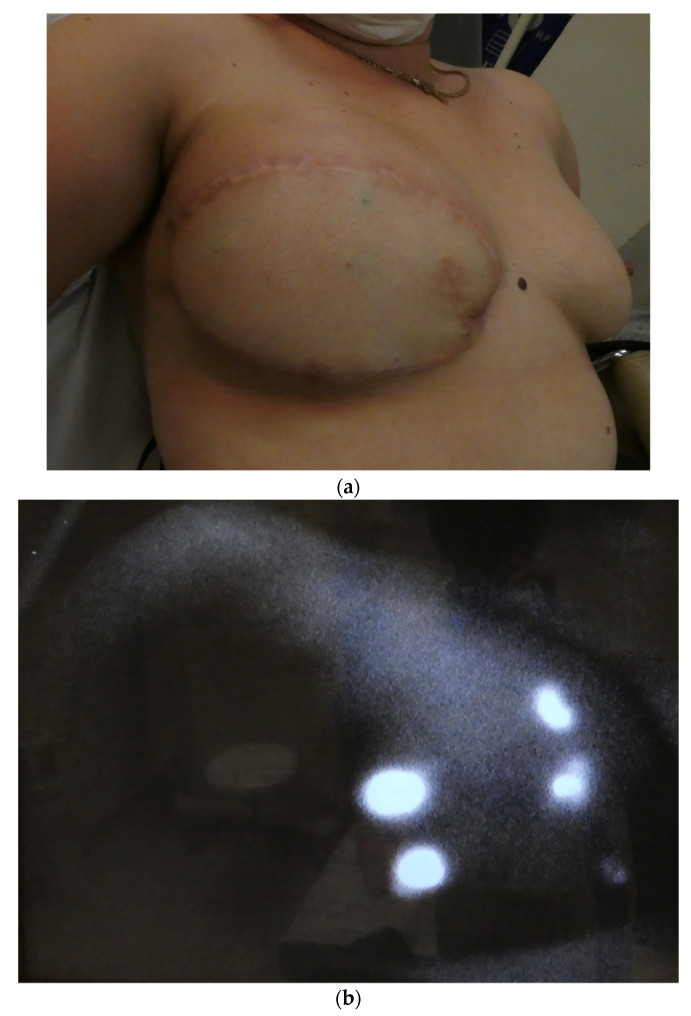
(**a**) DIEP flap breast reconstruction with classical design. (**b**) Breast reconstruction with classical design. No lymphatic flow restoration was observed at one year post-operatively.

All DIEP flaps were performed for breast reconstruction (Table 1). One of them was designed following the lymph axiality. The rest of DIEP flaps were designed upside down with the navel in the inframammary fold. Three patients were previously treated by radiation therapy (RT). Two of them had lymph node excision. The only DIEP with lymph flow restoration was the one designed following the lymph axiality. This patient did not receive RT nor had lymph node excision. The lymphatic restoration was located in the contralateral mammary area. No other flap showed lymphatic restoration. Three cases of post-operative seromas were reported but no clinically evident flap lymphoedema was observed (Figure 4 and Figure 5).

## 4. Discussion

This pilot study aimed to show that spontaneous lymphatic flow restoration between flap and recipient site is possible in flaps without a lymphatic tissue transfer but is uncertain. In our study, cases with spontaneous lymph flow restoration were designed as recommended by Yamamoto et al. and when there were no local restricting factors such as radiotherapy. In those cases, it seemed like the ICG flow followed the initial lymphatic pathways of the flaps then the major pathways of the limb/thorax. The hypothesis that lymphogenesis and anastomosis occurs between lymphatic tissues of the flap and the recipient site is the most relevant.

Our method is different from the Yamamoto’s original method in which ICG dye was injected in the distal region to the flap. By our method, only lymph drainage from the flap to the proximal region can be visualized. The reason why it has been decided to inject ICG directly in the flap was to avoid any confusion between a possible spontaneous lymphatic bypass deeper or lateral to the flap and the flap lymphatic pathway. The limitation of this method is that we could not prove if the lymphatic pathway of the distal part of the limb spontaneously anastomosed with the lymphatic pathway of the flap. Further, some patients needed orthopedic revision surgeries with distal incisions through the distal lymphatic pathway. In post-traumatic or oncologic limb reconstruction, ALT flap is the preferred one in our center. ALT flap is a large flap, reliable, easily dissected, with a relatively large pedicle and has a direct donor site suture. In most of the cases, an ALT flap is designed as the limb axis because of the location of the donor pedicle. This characteristic makes the preservation of the lymphatic axiality possible. Two of the seven ALT flaps were designed according to the defect and the lymph axiality. The other five flaps were only designed according to the defect, but they were placed following the limb axis and the same direction (proximal to proximal). The ALT case secondary to the osteosarcoma was the only one presenting a hypertrophic scare. Extended fibrosis has been described as a limitation of spontaneous anastomosis. Despite the scare and the previous radiotherapy, spontaneous lymphatic anastomosis was observed. The conventional DIEP flap used for breast reconstruction does not include inguinal lymph node. In our center, the flap is designed upside down, with the umbilicus in the infra mammary fold and the deep inferior epigastric pedicle is anastomosed with internal mammary pedicle [30,31,32]. This design does not allow a perfect lymphatic restoration. As Felmerer et al. [33] described, superficial lymphatic vessels run vertically in the subcutaneous tissue and the deep lymphatic vessels follow the flap pedicle. The classical DIEP insertion in the recipient site does not respect the insertions rules described by Yamamoto et al. [28]. The failure of spontaneous lymph flow restoration would be also attributable to the recipient site radiotherapy which is absent in 6 ALT flaps cases. In our study, only one DIEP flap had a spontaneous lymph flow restoration to the contralateral thoracic area. The only DIEP with spontaneous lymph flow restoration was a case of a patient with small contralateral breast and a large flap allowing a flap design regarding the lymphatic pathways. Several factors such as venous lesion, secondary surgeries, osteosynthesis material and lymphatic interruption maintain a chronic edema which can affect the post-operative rehabilitation of patients [7,8,9,10,11,12,13,14,15,16,17,18,19,20,21,22,23,24,25,26,27,28,29,30,31,32,33,34,35,36,37,38,39,40,41,42,43,44,45,46,47,48,49,50,51,52]. When it appears, flap lymphedema progresses over time and is responsible for pathological states and affects patients’ quality of life. One major limitation of our study is that the effect of the surgery and flap reconstruction on lymphatic flow of the region cannot possibly be assessed by injecting ICG in the flap alone. Therefore, with this study we wanted to focus more on the lymphoedema of the flap and not on the lymphoedema of the recipient site. Our study shows that lymphatic flow restoration may prevent from chronic clinically evident flap lymphedema. Plastic surgeons should theoretically improve lymphatic flow of the affected area by designing the flaps in relation to the lymphatic pathways whenever possible. Lymphaticovenular anastomosis and lymph nodes transfer are both time consuming and can rarely be performed during the oncologic or post-traumatic surgery [34,35,36,37,38]. Further, both surgeries must be performed by highly trained surgeons and have possible outcomes. Further prospective studies are required to determine if the lymph axiality prevents from lymphedema and which flap is more effective in lymphatic drainage.

## 5. Limitations of the Study

As stated above, one major limitation of our pilot study is that the effect of the surgery and flap reconstruction on lymphatic flow of the region cannot possibly be assessed by injecting ICG in the flap alone. Therefore, with this study we wanted to focus more on the lymphoedema of the flap and not on the lymphoedema of the recipient site.

Furthermore, there are low patient numbers and two radically different scenarios: ALTs for lower limb and DIEPS for breast. Further, all ALTs were designed according to lymph axiality and only one DIEP was. Radiation therapy in almost half the cases makes the number per comparable groups even smaller. So, it is hard to really draw conclusions from such a heterogenous groups. For example, ALTs designed not following lymph axiality could also regenerate spontaneous linear lymphatic drainage, and one single case of DIEP designed according to axiality does not provide sufficient evidence to really support our hypothesis. For all these reasons, we are including more patients to provide more cases and stronger evidence of spontaneous lymph flow restoration following free flaps planning according to lymph axiality.

## 6. Conclusions

The pre-operative planning of free flap in reconstructive surgery should consider the lymph axiality because it seems to improve spontaneous lymph flow restoration between flap and recipient site without any lymphaticovenular anastomose or lymph node transfer. However, before we can confirm this, further studies are needed to confirm this hypothesis.

In particular, further studies with a longer follow-up are needed to investigate the lymphatic connections between the flap and the recipient area.

## Figures and Tables

**Table 1:** Patients characteristics.

Case	Age	Gender	Etiology	Flap	Smoking	Radiotherapy	Previous Surgery	Axially Designed	Spontaneous Lymphatic Anastomosis
**1**	22	F	Osteosarcoma	ALT	No	Yes	Cancer excision	Yes	Yes
**2**	35	M	Trauma	ALT	No	No	Osteosynthesis	Yes	Yes
**3**	42	M	Trauma	ALT	No	No	Osteosynthesis	Yes	Yes
**4**	37	M	Trauma	ALT	No	No	Osteosynthesis	Yes	Yes
**5**	51	F	Trauma	ALT	No	No	Osteosynthesis	Yes	Yes
**6**	53	M	Trauma	ALT	No	No	Osteosynthesis	Yes	Yes
**7**	60	M	Trauma	ALT	No	No	Osteosynthesis	Yes	Yes
**8**	63	F	Breast cancer	DIEP	No	No	No	Yes	Yes
**9**	58	F	Breast cancer	DIEP	No	Yes	Lymph node excision	No	No
**10**	59	F	Breast cancer	DIEP	No	Yes	Lymph node excision	No	No
**11**	66	F	Breast cancer	DIEP	No	Yes	No	No	No
**12**	68	F	Breast cancer	DIEP	No	No	No	No	No
**13**	68	F	Breast cancer	DIEP	No	No	No	No	No
**14**	59	F	Breast cancer	DIEP	No	No	No	No	No
**15**	64	F	Breast cancer	DIEP	No	No	No	No	No
